# Simultaneous Imaging of Cerebrovascular Structure and Function in Hypertensive Rats Using Synchrotron Radiation Angiography

**DOI:** 10.3389/fnagi.2017.00359

**Published:** 2017-11-02

**Authors:** Liping Wang, Zhihao Mu, Xiaojie Lin, Jieli Geng, Ti Qiao Xiao, Zhijun Zhang, Yongting Wang, Yongjing Guan, Guo-Yuan Yang

**Affiliations:** ^1^Department of Neurology, Ruijin Hospital, School of Medicine, Shanghai Jiao Tong University, Shanghai, China; ^2^Med-X Research Institute and School of Biomedical Engineering, Shanghai Jiao Tong University, Shanghai, China; ^3^Department of Pathology and Pathophysiology, Faculty of Basic Medical Sciences, Kunming Medical University, Kunming, China; ^4^Department of Neurology, Renji Hospital, School of Medicine, Shanghai Jiao Tong University, Shanghai, China; ^5^Shanghai Synchrotron Radiation Facility, Shanghai Institute of Applied Physics, CAS, Shanghai, China; ^6^Department of Radiology, Ruijin Hospital, School of Medicine, Shanghai Jiao Tong University, Shanghai, China

**Keywords:** angiography, cerebral vessels, hypertension, synchrotron radiation, vascular elasticity

## Abstract

Hypertension has a profound influence on the structure and function of blood vessels. Cerebral vessels undergo both structural and functional changes in hypertensive animals. However, dynamic changes of cerebrovasculature and the factors involved in this process are largely unknown. In this study, we explored the dynamic changes of vascular structure in hypertensive rats using novel synchrotron radiation angiography. Twenty-four spontaneously hypertensive rats (SHR) and 24 Sprague–Dawley (SD) rats underwent synchrotron radiation (SR) angiography. Each group had 8 animals. We studied the cerebral vascular changes in SHR over a time period of 3–12-month and performed quantitative analysis. No vascular morphology differences between SHR and SD rats were observed in the early stage of hypertension. The number of twisted blood vessels in the front brain significantly increased at the 9- and 12-month observation time-points in the SHR compared to the SD rats (*p* < 0.01). The vessel density of the cortex and the striatum in SHR was consistently higher than that in SD rats at time points of 3-, 9-, and 12-month (*p* < 0.001). Vascular elasticity decreased both in SHR and SD rats with aging. There were statistically significant differences in the relative vascular elasticity of extracranial/intracranial internal carotid artery, middle cerebral artery, posterior cerebral artery and anterior cerebral artery between SHR and SD rats at 12-month (*p* < 0.01). We concluded that the dynamic vascular alterations detected by SR angiography provided novel imaging data for the study of hypertension *in vivo*. The longer the course of hypertension was, the more obvious the vascular differences between the SHR and the SD rats became.

## Introduction

Hypertension is a complex disease resulting from many pathogenic factors and has an impact on nervous system diseases. Studies showed that a long history of hypertension was the key risk factor for stroke (Takahashi et al., 2011; Turin et al., 2016). Hypertension could cause cerebral small vessel disease, which induced white matter damage and cognitive decline (Kaiser et al., 2014). Hypertension also impaired neurogenesis and long-term memory (Shih et al., 2016). Previous studies showed that cerebral vessels underwent both structural and functional changes in hypertensive animals (Nordborg and Johansson, 1980; Winquist and Bohr, 1983; Hughes and Bund, 2002; Cates et al., 2012). Cerebrovascular remodeling was the most important pathological change during hypertension (Lin et al., 1990; Lee and Griendling, 2008). For example, cerebral arteries from spontaneously hypertensive rats (SHR) were less compliant than those of the control. The arterial media was thicker during established hypertension in SHR (Mulvany et al., 1978; Nordborg and Johansson, 1980). Impairment of blood-brain barrier was induced by hypertension (Ueno et al., 2011; Biancardi and Stern, 2016). However, most of these studies were conducted using histology and immunohistochemistry in fixed brain or vascular tissue (Carnevale et al., 2012; Chan et al., 2015). Detecting dynamic changes of vascular morphology in living animals is a great challenge for neurologists and neurobiologists. Studies *in vitro* cannot detect the real pathological change of cerebral vessels. Current *in vivo* techniques for assessing vasculature and arterial functions still suffer from insufficient imaging resolution, making it difficult to detect subtle changes in biological specimens (Lin et al., 2013; Yuan et al., 2013). Little is known about the structural and functional changes of deep brain vessels in hypertensive animals (Morishita et al., 2006). Thus, developing a novel approach to dynamically and directly observe cerebrovascular alterations *in vivo* is necessary and timely, and could provide further insight into the pathology of vascular disorder in hypertensive animals.

Common devices used to visually study vascular morphology in living animals include optical imaging, CT angiography (CTA), MR angiography (MRA), digital subtraction angiography (DSA) and color Doppler ultrasonography (CDU) (Acar et al., 2015; Hoshikawa et al., 2016; Hsieh et al., 2016; Su et al., 2016; Xu et al., 2016; Chen et al., 2017). CTA and DSA are both x-ray based imaging methods used to assess vascular abnormalities in clinical and animal studies (Walkoff et al., 2016; Sun et al., 2017). High-resolution MRA is used to measure vessel patency in the entire process longitudinally (Duong, 2012; Shih et al., 2012). However, the resolution of these methods is limited for the detection of microvasculature disorders in rodents, as they have difficulty in detecting changes of small vessels less than 500 μm in diameter (Dehkharghani et al., 2015; Detorakis et al., 2015; Wu et al., 2015; Kampschulte et al., 2016). Synchrotron radiation (SR) x-ray has a wide continuous adjustability of energy, high brilliancy, narrow pulse, collimating coherence and monochromaticity (Hu et al., 2014; Zhang et al., 2014). The spatial resolution of SR x-ray imaging can reach sub-micron, around 1,000 times higher than that of conventional X-Ray absorption imaging (Liu et al., 2006; Zhang et al., 2014). Recent developed SR angiography showed that it could be used for high-resolution imaging of cerebral vasculature in rodents (Lin et al., 2013; Yuan et al., 2013; Xi et al., 2015; Mu et al., 2016). It provides a unique tool for monitoring real time hemodynamic changes in blood flow and microvascular morphology. Fuji et al. used SR angiography to explore the association between endothelial function and micro-vascular remodeling in pulmonary arterial hypertension (Fuji et al., 2016). Miya et al. made use of synchrotron radiation to visualize the renal arterioles, glomeruli, and proximal tubules of rats *in vivo* (Miya et al., 2017). By performing SR angiography, our previous studies demonstrated that netrin-1 overexpression in the mouse brain, and transplantation of endothelial progenitor cells promoted functional angiogenesis (Lu et al., 2012; Chen et al., 2014). In addition, SR angiography has also been used in the evaluation of cerebral vasospasm in subarachnoid hemorrhage and cerebrovascular changes in hyperglycemic rats (Cai et al., 2012; Mu et al., 2016).

In this study, we performed SR angiography in SHR and Sprague–Dawley (SD) rats to compare the differences both in vascular structure and function with aging. In consideration of parts where cerebral diseases such as stroke might occur, our study focused on the Willis circle, cortex and striatum (Lipton, 1999). We also explored whether alterations of the vascular structure were related to the blood pressure and age and thereby affected outcomes of vascular diseases.

## Materials and methods

### Animals and experimental groups

Animal procedures were reviewed and approved by the Institutional Animal Care and Use Committee (IACUC) of Shanghai Jiao Tong University, Shanghai, China. Adult male SHR (*n* = 24) and adult male SD rats (*n* = 24) weighing 250 ± 30 grams (Sippr-BK, Inc., Shanghai, China) were used in the study. The SHR were randomly divided into 3 groups of 8 referred to as 3-, 9-, and 12-month-old groups. The SD rats used as controls were randomly divided into 3 groups as well. Animals were fed normally in the SPF proved animal facility in Shanghai Jiao Tong University, China.

### Measurement of blood pressure

Systolic blood pressure (SBP) and diastolic blood pressure (DBP) were measured every week using a non-invasive tail cuff acquisition method (BP-98A, Softron Beijing Incorporated, Beijing, China). Rats were placed in a restrainer to acclimate themselves. All rats were first acclimated to the blood pressure measurements for 3 days before the acquisition (Biancardi et al., 2014). Arterial blood pressures were derived from the average results of >3 measurements in each recording session. Both SBP and DBP were obtained and recorded for the data analysis.

### SR angiography

SR angiography was conducted at the X-ray imaging beam line BL13W in Shanghai Synchrotron Radiation Facility (SSRF). Imaging setup and procedures have been described previously (Guan et al., 2012; Yuan et al., 2014). Briefly, the average beam current was 145 mA, and X-ray energy was 33.2 keV. Animals were anesthetized by using ketamine/xylazine (100 mg/10 mg/kg, intraperitoneally) during the imaging process. An angiographic tube formed by connecting a PE-10 tube with a PE-50 tube was inversely inserted into the external carotid artery to allow injection of a contrast agent. The rat was placed perpendicular to the beam on its left side, and 160 μl of diluted iodinated contrast medium, ipamiro (General Electric Company, Shanghai, China), was injected into the common carotid artery through the angiographic tube at a speed rate of 6 ml /min using an automated micro-syringe pump (LSP01-1A, Longer; Baoding, China). An X-ray complementary metal oxide semiconductor (pixel size 13.0 × 13.0 μm, frame frequency 30 Hz, Hamamatsu Ltd., Japan) was used to record high-resolution real-time angiographic images. The X-ray complementary metal oxide semiconductor was positioned at a distance of 780 mm from the sample.

### Assessment of vessel morphology

We used image analysis software (Image J, National Institutes of Health, Bethesda, MD) to conduct measurements of the internal diameter of vessels in the circus Willis. Vessel internal diameter presented was the average of three independent measurements. To assess cortical and striatal microvascular perfusion, we measured the vessel density using a perfusion angiogram. A specialized program written in Matlab (MathWorks, Natick, Massachusetts) was used to identify perfused vessels from original SR angiography images. The vessel signals in each area were summed.

To assess the number of curly small vessels, we selected the same area on each image and amplified the area to find bending parts of the small vessels, which presented high signal value, then calculated the number of the bending parts of each group.

### Calculation of arterial elasticity

The theory and method of the elasticity calculation has been described previously (Lin et al., 2015). In the imaging procedure, sequential x-ray transmission images *I*(*x, y, t*) of the rat brain were recorded. Relative vascular elasticity was estimated from the absorption maps *I*_*abs*_(*x, y, t*).

(1) $$\begin{array}{r} I_{abs}\left(x,y,t\right)=\text{ln}\left(I_{abs}\left(x,y,0\right)\right)-\text{ln}\left(I_{abs}\left(x,y,t\right)\right) \end{array}$$

The blood vessels were then manually segmented, and the diameter r_0_ of each vessel was calculated for each ridge point. The absorption data of the cross-sectional line at each ridge point in *I*_*abs*_(*x, y, t*) was extracted based on the perpendicular relationship between the ridge and cross-section. The spatio-temporal dynamics *I*_*abs*_(*x, y, t*) of each cross-section were constructed from the image sequences *I*_*abs*_(*x, y, t*). In this study, the spatio-temporal dynamics of the contrast agent in *I*_*rt*_(*r, t*) (*t* < *t*_0_*)* were fitted to a Gaussian surface (Equation 2).

(2) $$\begin{array}{r} I_{rt}\left(r,t\right)=a\cdot e^{-\left({\left(r-r_{0}/2\right)}^{2}/\sigma_{r}^{2}+{\left(t-t_{0}\right)}^{2}/\sigma_{t}^{2}\right)}+b \end{array}$$

After estimating the fitting parameters in Equation (2), the relative velocity v of each cross-section was calculated using Equation (3).

(3) $$v=\int_{0}^{t_{0}}\int_{-\frac{r_{0}}{2}}^{\frac{r_{0}}{2}}\frac{I_{rt}\left(r,t\right)}{{\text{t}}_{0}r_{0}}drdt$$

The elasticity E of each cross-section was calculated using Equation (4).

(4) $$\begin{array}{r} E=\frac{v}{r_{0}} \end{array}$$

### Hematoxylin and eosin staining

Rats were anesthetized after SRA and perfused with saline followed by 4% paraformaldehyde transcardially. The brains were fixed in 4% paraformaldehyde for 24 h and dehydrated with a graded alcohol system and then embedded in paraffin. A series of 8-μm-thick coronal sections were prepared for hematoxylin and eosin staining according to a previously reported study (Lin et al., 2013).

### Statistical analysis

Data were presented as mean ± standard deviation (SD). Blood pressure, the number of curly vessels, and vascular elasticity among different groups were compared with an unpaired Student *t*-test using SPSS 16.0 (SPSS Inc., Armonk, NY). One-way ANOVA was carried out to assess within-group differences of arterial elasticity, followed by a 2-tailed Student *t*-test to determine statistical significance. A probability value of *p* < 0.05 was considered significant.

## Results

### Sustained hypertension in SHR over time

**Figure 1:**
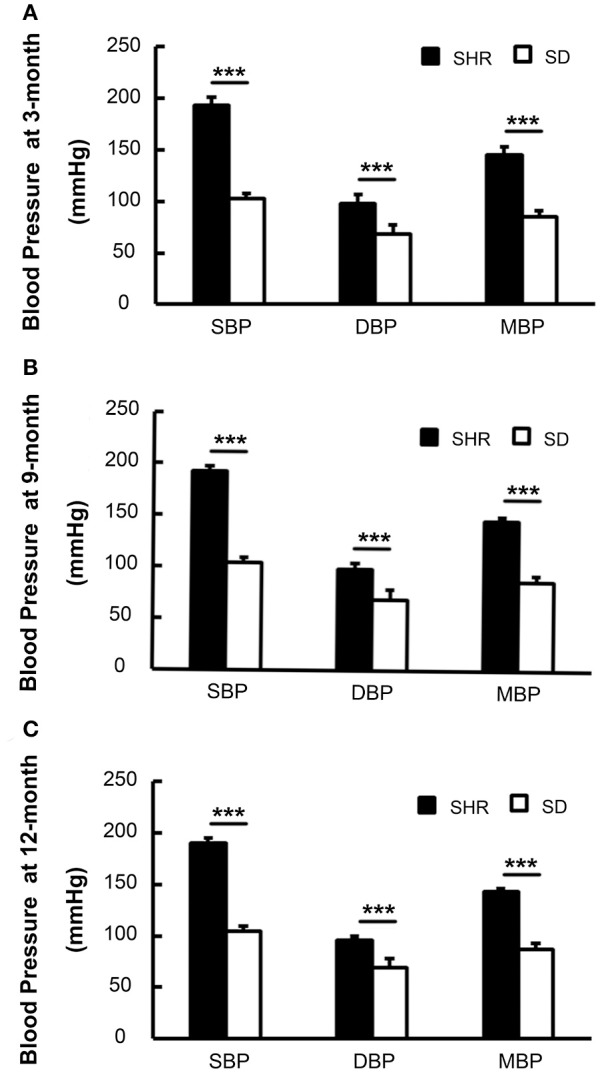
Blood pressure of SHR was higher than SD rats in 3-**(A)**, 9-**(B)**, and 12-month **(C)**. SBP, systolic blood pressure; DBP, diastolic blood pressure; MBP, mean blood pressure. ^***^p < 0.001.

We measured the blood pressure of both SHR and SD rats. Blood pressure in SHR and SD rats both remained stable from the 3rd−12th month. Meanwhile SBP, DBP, and mean blood pressure (MBP) were significantly elevated in the SHR 3-, 9-, and 12-month groups compared to the age-matched SD rats (*p* < 0.001). The mortality of SHR was less 10% at the 12-month observation point. This demonstrated that the blood pressure of the SHR we used in the study was at a relatively stable level (Figure 1), and thus this animal model was suitable for the vascular study.

### Increase of curly small vessels in SHR

**Figure 2:**
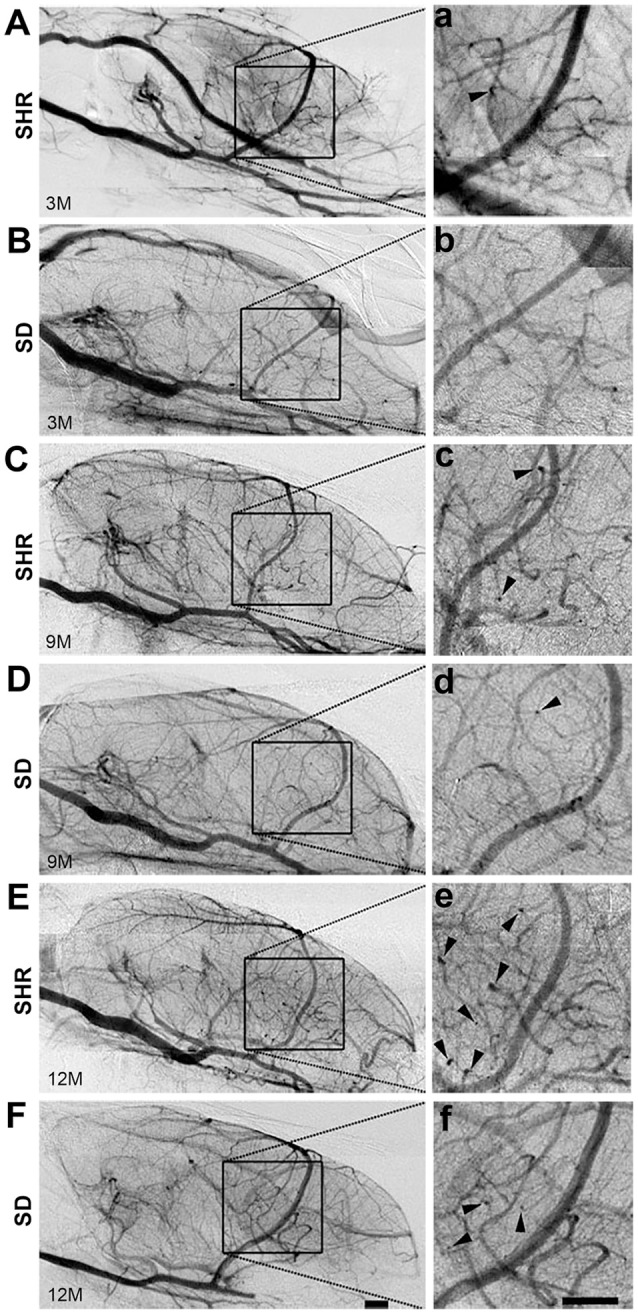
Curly vessels in the brain of SHR increased significantly than SD rats. Angiography pictures were presented from SHR **(A,C,E)** and SD **(B,D,F)** rats in 3-, 9-, and 12-month. The images in square frames of graphs **(a–f)** were amplified as images **(A–F)**, respectively. Square frame defines the selected area for calculating. Arrowhead points to curly vessels. Scale bar = 1 mm.

**Figure 3:**
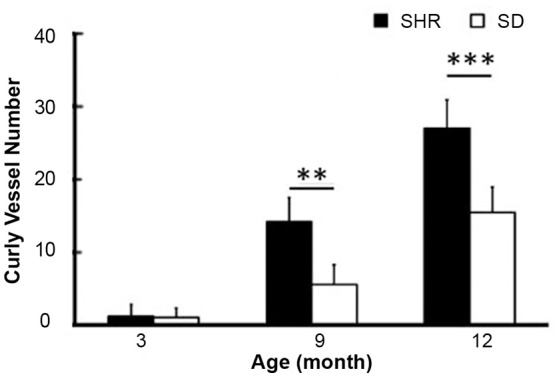
Bar graph of curly vessel number. Data are mean ± SD, *n* = 6 per group, ^**^p < 0.01, ^***^p < 0.001.

To study the dynamic effect of hypertension on small intracranial vessels, we performed SRA to explore the structural alterations of the cerebral vessels. We found that the morphology of small vessels in the striatal area became disordered. Twisted small vessels manifested, and the curvature of the terminal branches significantly increased with time (Figure 2). We semi-quantified the number of intracranial curly small vessels in the brains of SHR and SD rats. The results showed that there was no significant difference in the number of curly small vessels between SHR and SD rats in the 3-month groups (1.24 ± 1.55 vs. 1.12 ± 1.23, *p* > 0.05). However, when hypertension was sustained for 9 months, the number of curly small vessels in the brain of SHR was significantly increased compared to those in the SD rats (Figure 3, 26.93 ± 3.86 vs. 15.38 ± 3.46, *p* < 0.001). Similarly, an increase of vascular curvature was not evident in the early stage of hypertension. In later hypertension this change became obvious, and the quantity of the curly small vessels increased with time.

### Increased vascular density in SHR

**Figure 4:**
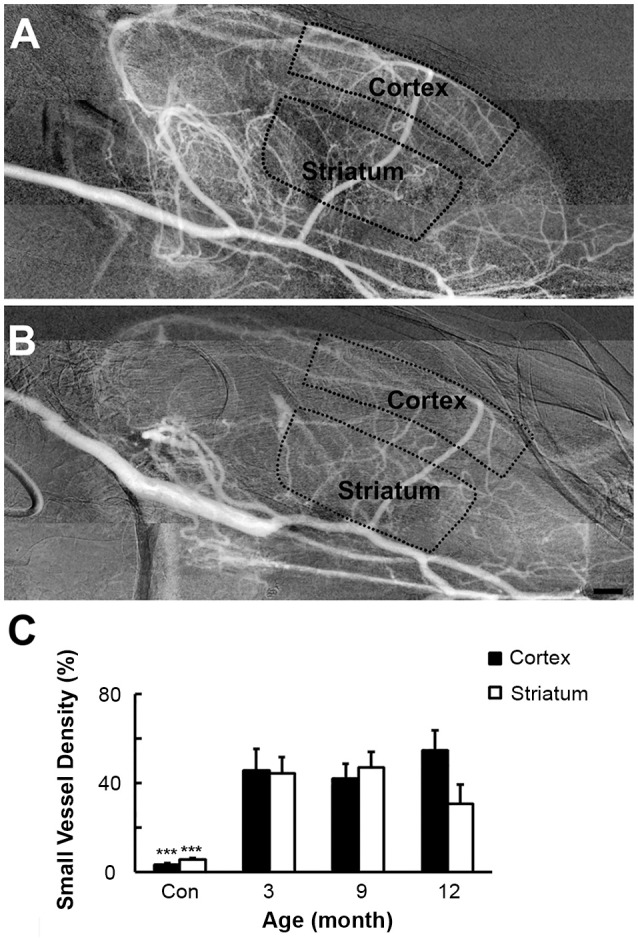
Vascular density of SHR and SD rats. SHR **(A)** had increased perfused small vessel density in cortex and striatum than SD rats **(B)** at 3-, 9-, and 12-month. **(C)** Bar graph of small vessel density. Bar = 1 mm. Data are mean ± SD, *n* = 6 per group, ^***^p < 0.001.

Using the Matlab software, we calculated the vascular density in the cortex and striatum from the original angiographic images. Perfused small vessel density in the cortex (45.6 ± 9.7, 41.9 ± 6.7, and 54.5 ± 9.1% vs. 3.5 ± 0.5%) and striatum (44.4 ± 7.3, 47.1 ± 7.0, and 30.7 ± 8.6 vs. 5.6 ± 0.8%) of SHR was consistently higher in the 3-, 9-, and 12-month groups than that in the SD rats (Figure 4, *p* < 0.001). Cerebral microcirculation perfusion volume was increased in SHR, but there was no difference in microcirculation perfusion in different time groups of SHR, indicating that hypertension could not further affect the microcirculation blood flow. Enhanced perfused small vessel density could be the result of an increase in the number of open small vessels, induced by hypertension.

### Reduction of vascular elasticity with increased duration of hypertension

**Figure 5:**
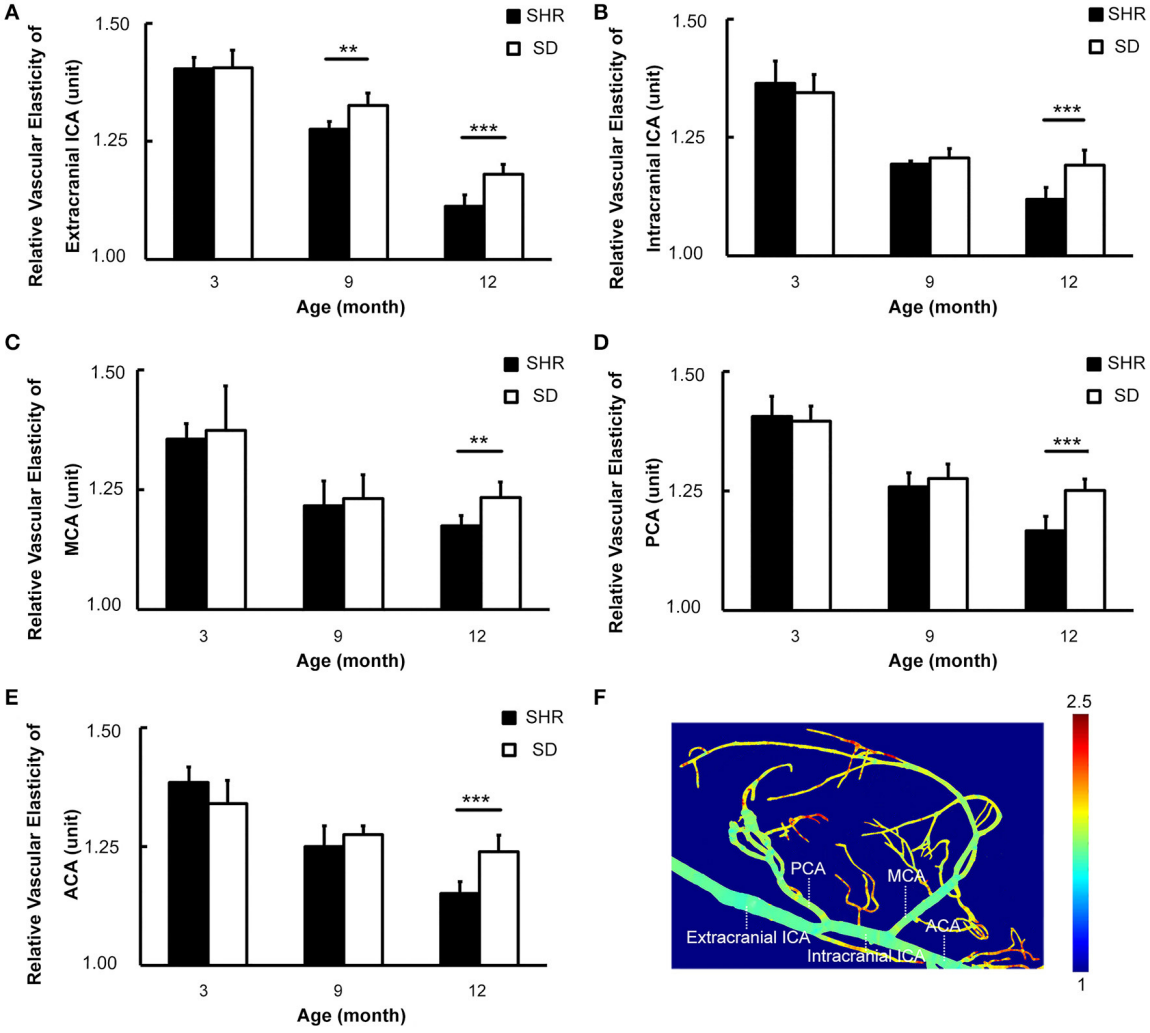
Circus Willis of SHR had decreased vascular elasticity with the duration of hypertension increased. **(A–E)** Bar graph of relative vascular elasticity of extracranial ICA, intracranial ICA, MCA, PCA, and ACA in 3-, 9-, 12-month respectively. **(F)** Pseudocolor image demonstrated the vascular elasticity in extracranial ICA, intracranial ICA, MCA, PCA, and ACA. Data are mean ± SD, *n* = 6 per group, ^**^p < 0.01, ^***^p < 0.001.

To investigate the dynamic effects of hypertension on the intracranial large vessels, we calculated the vascular elasticity of all branches of Circus Willis using functional SR angiography. The elasticity values were normalized for comparison. The results showed that the vascular elasticity of SHR and SD rats decreased with aging, but there was a greater decrease in the SHR compared to the SD rats. The relative vascular elasticity of extracranial ICA of the SHR was lower than that of SD rats at the 9-month time point (0.28 ± 0.02 vs. 0.33 ± 0.03, *p* < 0.01). There were no significant differences in relative vascular elasticity of extracranial ICA, intracranial ICA, middle cerebral artery (MCA), posterior cerebral artery (PCA) and anterior cerebral artery (ACA) between the SHR and the SD rats at 9-month. It was noted that the differences of extracranial ICA, intracranial ICA, MCA, PCA and ACA between the SHR and the SD rats were significant at 12-month (Figure 5, 0.11 ± 0.02 vs. 0.18 ± 0.02, 0.12 ± 0.03 vs. 0.19 ± 0.03, 0.18 ± 0.02 vs. 0.23 ± 0.03, 0.17 ± 0.03 vs. 0.25 ± 0.02, 0.15 ± 0.03 vs. 0.24 ± 0.03, *p* < 0.001). Our results suggested that reduced vascular elasticity was a long-term effect of hypertension but did not occur after a short period of hypertension, as in the early stage of hypertension vascular elasticity was not affected. We only began to detect impairment in vascular elasticity caused by hypertension after the 9th month.

### Hypertension induced arterial stenosis in ICA

**Figure 6:**
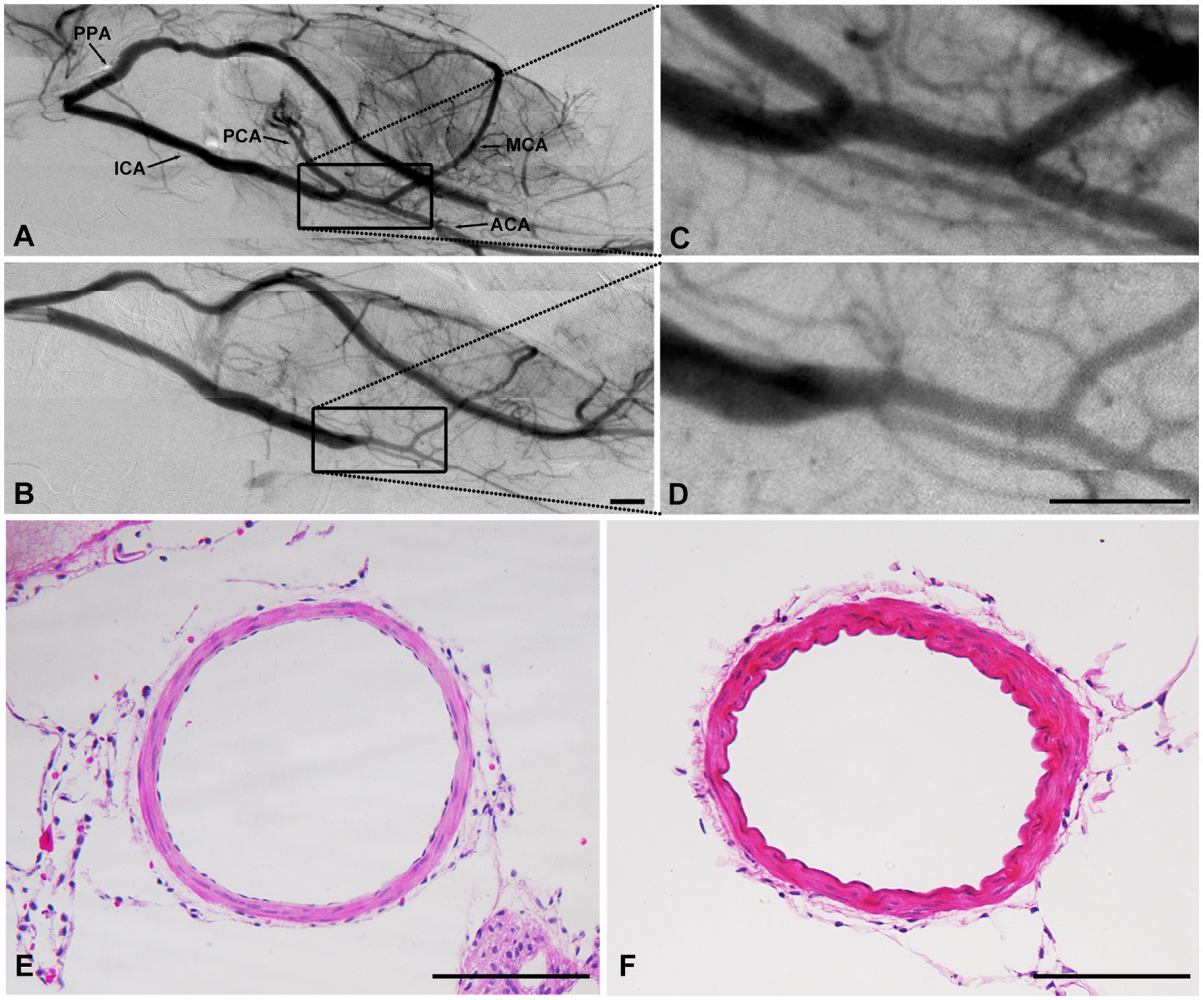
SHR had arterial stenosis in ICA. Cerebral vascular morphology imaged by SR angiography in SHR **(B)** and SD rats **(A)**. **(C,D)** were higher amplification. HE result showed that the diameter decreased while the wall-lumen ration of ICA increased in SHR **(F)** than that in SD rats **(E)**. **(A–D)**: Scale bar = 1 mm. **(E,F)**: Scale bar = 100 μm.

Interestingly, comparing the diameter of arteries between the SHR and the SD rats, we found that the diameter of the intracranial ICA but not the extracranial ICA, MCA or ACA, was narrowed in SHR in SR angiography images (Figure 6, 0.10 ± 0.04 vs. 0.19 ± 0.05, *p* < 0.01). The diameter of the ICA did not recover over the duration of study, suggesting that this phenomenon of intracranial ICA stenosis was permanent.

## Discussion

Using novel SR angiography technique, we first demonstrated dynamically in living SHR that: (1) the morphology of cerebral vessels gradually became twisted and multicurled with hypertension; (2) small vessel density was increased in the forebrain of SHR compared to that in the SD rats; (3) stenosis appeared in the intracranial ICA in SHR; and (4) the vascular elasticity of the branches in Willis Circus decreased with hypertension.

Using SR angiography, a unique technique in the imaging field, we showed dynamic vascular structural alteration induced by hypertension *in vivo*. We found that the vascular structure became abnormal in the forebrain, especially in the MCA territory, with hypertension. There were obviously more tortuous vessels, and increased curvature of terminal branches in the SHR. Though there was no significant difference between SHR and SD rats at 3-month, the number of curly vessels at 9-, and 12-month was significantly increased in the SHR compared to the SD rats. The development of hypertension in the SHR had different effects on the vascular resistance of different parts of the vascular bed (Faraci and Heistad, 1990). The large arteries were affected at the early stage of hypertension. When hypertension was maintained at a certain level for a period of time, small and micro arteries carried more vascular resistance (Bohlen, 1989). Morphological abnormality in the SHR indicated that small cerebral vessels were affected by sustained hypertension. These multicurled small vessels could affect the function of cerebral blood circulation and thus further impact on the development and prognosis of vascular disorder, contributing to the occurrence of stroke.

SR angiography results also showed that small vessel density was increased in the cortex and striatum of the SHR compared to those of the SD rats at 3-, 9-, and 12- month. Vessel density is an indicator of microvascular perfusion. Immunohistochemical staining of cerebral blood vessels could only reflect the number of blood vessels, not the exact number of the perfused microvessels. SR angiography is the only way to present cerebral microcirculation perfusion.

Data analysis suggested that hypertension could increase the functional capacity of capillaries. However, whether this increased capillary capacity could alleviate the insufficient blood supply during ischemic stroke needs to be further explored. Finally, we found intracranial ICA stenosis in SHR, suggesting that the intracranial ICA underwent reconstruction to adapt to hypertension. Remodeling induced by hypertension contributes to decreased diameter and increased wall-lumen ratio of vessels (Mulvany et al., 1996; Bonacasa et al., 2008; Kumai et al., 2008; Pires et al., 2010), which could reduce cerebral blood flow after ischemic stroke and affect recovery.

The functional alteration of cerebral vessels was induced by hypertension. Vascular elasticity is an indicator of vascular function (Fabiani et al., 2014; Turk et al., 2015). The functional status of the vasculature could therefore be predicted by vascular elasticity. We found that vascular elasticity of the branches in Circus Willis decreased with sustained hypertension. We noted that in the early stage of hypertension, there was no difference in vascular elasticity between the SHR and the SD rats. However, decreased vascular elasticity was associated with hypertension. Moreover, reduced vessel elasticity could cause vascular wall stiffness, which is the most important factor for hemorrhagic stroke.

Functional SR angiography technique provides a novel non-invasive technique for detecting vascular elasticity. Compared to other methods of measuring cerebral vascular elasticity, functional SR angiography produces less damage (Regrigny et al., 2000). The method we developed could be used to monitor dynamic vascular function. Structural and functional alterations could be viewed in a single contrast image. Dynamic measurement of vascular elasticity *in vivo* is characteristic of functional SR angiography. This method is applicable to the measurement of deep blood vessels, which could not be detected by ultrasound elastography (Li et al., 2016). Although MRI is suitable for *in vivo* imaging, the lower resolution limits its use in the detection of small vessel changes. The slow imaging speed of MR is not suitable for dynamic imaging in living animals (Hughes et al., 2015). At the same time, we also showed that SR angiography could achieve real-time dynamic monitoring of vascular morphology in the rodent brain. We did not detect any difference in vasculature between the SHR and the SD rats using MRI and MRA (data not shown), which supported the fact that MRA was not a sensitive enough approach when exploring the structural and functional changes in cerebral vessels due to hypertension because of the limited resolution (Yang et al., 2008; Guan et al., 2012).

There were some limitations in our study. A previous study showed that aging is also associated with changes in vascular structural and cellular function in the brain (Chowdhury et al., 2011). As most clinical hypertension patients are older than 65 years old (Marini et al., 2001), researches on aged rats or mice are of great importance. In our study, we only explored vascular changes of SHR at 3-, 9-, and 12-month. Three-month represented the early stage of hypertension. The 6-month time point was skipped because the data of this time point resembled that of the 9-month time point in the preliminary study. The vascular changes of SHR at 24-month which could be referred to as aged rats still need to be explored (Tang et al., 2014). Compared to other studies using 1–5 months old animals, our study lasted longer (Coyle and Jokelainen, 1983; Winquist and Bohr, 1983; Kitamura et al., 2016; Stanzione et al., 2017; Takesue et al., 2017). Besides, the relationship between the vascular alterations including multicurled vessels, vessel density, vascular elasticity, and prognosis of stroke remains unclear. The molecular mechanism of vascular changes needs to be further investigated.

Deeper mechanisms of structural and functional alterations of cerebral vessels in hypertension remain to be studied further. Our *in vivo* dynamic study of living SHR at different stages of age could contribute to hypertension research. Hypertension can often cause pathological changes in the cerebrovasculature, and therefore monitoring intracranial vessel morphology and vascular function appears to be extremely important. SR angiography has great potential for experimental application, as this technique of fast simultaneous imaging of vascular morphology and elasticity can help to evaluate vascular function status.

In conclusion, our data directly demonstrated the structural and functional alterations of cerebral vessels induced by hypertension *in vivo*, indicating that functional SR angiography is a unique tool for detecting vascular disorder in rodents, which may benefit both clinical diagnosis and therapy.

## Author contributions

LW and ZM contributed to the design of experiment and the draft of the manuscript. XL, JG, and ZZ contributed to the acquisition of the data. TX, YW, YG, and GY helped to design the study and revise the manuscript. All authors approved the final version of the manuscript.

### Conflict of interest statement

The authors declare that the research was conducted in the absence of any commercial or financial relationships that could be construed as a potential conflict of interest.

## Abbreviations

ACA: anterior cerebral artery
CDU: color Doppler ultrasonography
CTA: CT angiography
DBP: diastolic blood pressure
DSA: digital subtraction angiography
ICA: internal carotid artery
MBP: mean blood pressure
MCA: middle cerebral artery
MRA: MR angiography
PCA: posterior cerebral artery
SBP: systolic blood pressure
SD rats: Sprague–Dawley rats
SHR: spontaneously hypertensive rats
SR: synchrotron radiation.
